# Evaluation of high-fidelity and virtual reality simulation platforms for assessing fourth-year medical students’ encounters with patients in need of urgent or emergent care

**DOI:** 10.1080/07853890.2024.2382947

**Published:** 2024-07-30

**Authors:** Matthew Malone, David P. Way, Cynthia G. Leung, Douglas Danforth, Kellen Maicher, Joanne Vakil, Nicholas Kman, Christopher San Miguel

**Affiliations:** a Department of Emergency Medicine, OH State University College of Medicine, Columbus, OH, USA; b Department of Obstetrics & Gynecology, Ohio State University College of Medicine, Columbus, OH, USA; c James Cancer Hospital, Ohio State University, Columbus, OH, USA; d Office of Curriculum and Scholarship, Ohio State University College of Medicine, Columbus, OH, USA

**Keywords:** Medical education, undergraduate, educational assessment, entrustable professional activity, virtual reality, educational, emergency medicine, critical care

## Abstract

**Background:**

Medical students in the U.S. must demonstrate urgent and emergent care competence before graduation. Urgent and emergent care competence involves recognizing, evaluating and initiating management of an unstable patient. High-fidelity (HF) simulation can improve urgent and emergent care skills, but because it is resource intense, alternative methods are needed.

**Study Objective:**

Our primary purpose was to use program evaluations to compare medical student experiences with HF and virtual reality (VR) simulations as assessment platforms for urgent and emergent care skills.

**Methods:**

During their emergency medicine clerkship, students at The Ohio State University College of Medicine must demonstrate on HF manikins, competence in recognizing and initiating care of a patient requiring urgent or emergent care. Students evaluated these simulations on a five-point quality scale and answered open-ended questions about simulation strengths and weaknesses. Faculty provided feedback on student competence in delivering urgent or emergent care. In 2022, we introduced VR as an alternative assessment platform. We used Wilcoxon Signed Ranks and Boxplots to compare ratings of HF to VR and McNemar Test to compare competence ratings. Comments were analyzed with summative content analysis or thematic coding.

**Results:**

We received at least one evaluation survey from 160 of 216 (74.1%) emergency medicine clerkship students. We were able to match 125 of 216 (57.9%) evaluation surveys for students who completed both. Average ratings of HF simulations were 4.6 of 5, while ratings of VR simulations were slightly lower at 4.4. Comments suggested that feedback from both simulation platforms was valued. Students described VR as novel, immersive, and good preparation for clinical practice. Constructive criticism identified the need for additional practice in the VR environment. Student performance between platforms was significantly different with 91.7% of students achieving competence in HF, but only 65.5% in VR (*p*≤.001, odds-ratio = 5.75).

**Conclusion:**

VR simulation functions similarly to HF for formative assessment of urgent and emergent care competence. However, using VR simulation for summative assessment of urgent and emergent care competence must be considered with caution because students require considerable practice and acclimation to the virtual environment.

## KEY MESSAGES

- Medical students found value in using virtual reality simulation as a platform for practice and feedback in a formative assessment arrangement.
- Students described the virtual reality simulation as immersive and good preparation for clinical practice.
- Technical difficulties were common and the student learning curve for acclimating and learning how to function in the virtual environment was noteworthy.

## Introduction

Widespread adoption of competency-based medical education has compelled the development of conceptual frameworks to guide the work of medical educators as they formulated competencies or outcomes of the medical education process [1]. New terminology, including competencies, entrustable professional activities, and milestones have been adopted to describe the work of a physician, and the knowledge and skills required of a medical learner [2]. In the United States, Entrustable Professional Activities (EPAs) emerged as the preferred framework for describing the discrete clinical tasks that medical students needed to be able to perform as they made the transition from medical school to residency [3]. Once a medical learner has demonstrated competency for a particular task, they are “entrusted” to perform that activity in the clinical environment with minimal supervision [4].

The first set of core EPAs were proposed by the Association of American Medical Colleges (AAMC) in 2014 [3]. These EPAs represented the essential clinical tasks that all medical students are expected to competently perform upon entry into residency. Since then, a number of other nations, such as Australia-New Zealand, Sweden, Canada, Switzerland, among others, have adopted similar guidelines for undergraduate medical education (UME) [5–8]. Notably, nearly all of these national guidelines include an EPA that involves the recognition and care of the emergent patient. The AAMC refers to this EPA as EPA-10, which requires medical learners to “recognize a patient needing urgent or emergent care and initiate proper evaluation and management” [4]. Subsequently, medical schools in the U.S. and elsewhere are charged with certifying that their medical graduates are competent to recognize a seriously ill or injured patient; call for help from a more experienced physician or mobilize resources; gather clinical data to guide treatment; and begin initial resuscitative measures including basic life support.

Earlier studies suggested that fourth year medical students struggled to demonstrate competence in assessing and managing unstable patients. Medical educators found that these students were less prepared to manage a simulated emergent diagnosis when compared to managing a range of non-acute conditions [9–12]. Further complicating the entrustment of medical students on EPA-10 is their lack of access to critically ill or injured patients in the emergency department [10].

While some EPAs might be amenable to work-based assessment, it is untenable to think that medical students could be safely assessed on independently evaluating and managing a seriously ill patient in the clinical setting [10–12]. Subsequently, high-fidelity (HF) human physiology simulation equipment (simulators or manikins) has been developed for simulation-based teaching and assessment of medical students on the skills related to EPA-10 [10, 13, 14]. Since realism is at the heart of HF simulation, it typically involves the recreation of the clinical environment in which care of the patient takes place. So when teaching and assessing learners to care for the seriously ill, this involves not only the replication of a seriously ill patient, but also the replication of the care environment such as an emergency department or resuscitation bay.

The community of medical educators and simulationists have referenced numerous learning theories to bolster their reliance on realism when developing simulations for teaching and learning medical skills such as EPA 10. These include constructivism (Vygotsky), experiential learning (Lewin and Kolb), and adult learning theory (Knowles) [15]. With regard to simulations specifically for teaching and learning the complex competencies related to EPA-10, the care of the seriously or critically ill or injured patient, we found a more suitable conceptual framework called Brain-based Learning (BBL) Theory [16]. Three foundational elements of Brain-based Learning support the use of simulation for teaching and learning the complex series of clinical tasks required when caring for a seriously ill patients, (which, for simplicity, we will refer to as “EPA-10 skills”): relaxed alertness, orchestrated immersion in realistic experiences, and active processing of experience.

Relaxed alertness suggests that simulation environments must be immersive and realistic, but low stress such that the fear of failure is removed. Realism of the simulation not only helps the learner engage with the emotions associated with the content to be learned; but also helps the learner to adapt between focused and peripheral attention, process both overt and covert knowledge, and engage both spatial and rote learning memory systems. Orchestrated immersion proposes that the simulation must involve more than just the brain but needs to engage the whole person. Finally, active processing suggests that Brain-based Learning supports the unique learning requirements of the individual learner theorized by other learning theories such as constructivism (Vygotsky), or multiple intelligences (Gardner) [15].

While effective for training and assessing medical learners on EPA-10 skills, HF simulation equipment along with the qualified technical support staff required to recreate a realistic analog setting of an emergency department is a significant institutional monetary investment [15]. The total upfront base cost of a high-fidelity programmable simulation mannequin capable of replicating the conditions of a seriously or critically ill or injured patient ranges from $65,000–$85,000, which does not include the cost of the table, gurney or bed to hold the mannequin, nor the cost associated with medical equipment, such as monitors, required to create a realistic scenario [15, 17, 18]. Subsequently, the problem associated with HF simulation is related to availability and associated costs that put it out of reach for many medical schools. In comparison, the total upfront base cost of our EPA-10 VR simulator, including the simulation software, custom conversion of cases into the software platform, the desktop gaming system and two virtual reality headsets was approximately $15,000. This simulation also requires physical space, about the size of a small classroom and a wireless internet connection (network or hotspot).

Virtual reality (VR) is an alternative platform for training and assessing future physicians on EPA-10. The potential advantages of VR based simulation education are extensive. VR simulators are already being used to educate and assess emergency medicine specialists, surgeons and other healthcare subspecialists in complex procedures that are too dangerous to practice on live patients [19]. VR advocates have noted that virtual standardized patient simulations can reduce cost, faculty time and resources needed to assist students in developing their clinical skills [20]. VR interactions can be standardized across students and can potentially provide a more accessible opportunity for students to practice skills needed for treating seriously ill patients in a safe, nonthreatening environment [21].

Another advantage of the VR simulation platform is its suitability for simulation-based master learning, which provides learners the opportunity to practice under direct observation and to receive timely feedback to master skills [22–24]. Traditional standardized patients and high-fidelity simulated patients may involve direct observation by faculty to generate feedback, however VR platforms can provide direct, real time, automated feedback [19]. Finally, VR platforms provide “gaming type mechanics” that allow learners to track progress and transform complex cognitive tasks into a series of simpler steps [25]. They can also be programmed to monitor learner performance and automatically adapt the difficulty level of scenarios and tasks as the learner’s progress through various levels of proficiency until they reach mastery levels of performance [19].

Virtual Reality is not without its own limitations. One of the major downfalls of VR platforms is the lack of realistic tactile sensation and haptic feedback [26]. Haptics are very important in procedural simulations and allow learners to develop muscle memory such as how much force to apply to a scalpel or chest compressions. This is less of a limitation when the specific simulation case does not involve medical procedures. While in the long run, the cost of VR is much less expensive than the cost of high-fidelity simulation, however startup costs for VR can be substantial. Hardware costs such as VR screens, software or head-mounted displays may be cost prohibitive for some institutions, which may limit the widespread adoption of VR training and assessment. However, as mainstream gaming becomes more popular and available, the cost of these technologies has been declining, making them more accessible to medical educators [15].

The literature on the use of virtual reality for medical education began to flourish in the last decade [27–28]. However, in preparation for this project, our literature search yielded very few articles which made comparisons between HF and VR simulation platforms specifically for assessing medical student competence in caring for seriously ill or injured adult patients (EPA-10) [29–31]. The exception was an investigation by McNamara, et al. who gathered evaluation data from a small group of 20 students during their final year of medical school in order to profile their encounters with simulation. The article describes medical student experiences with both HF and VR simulation for assessing competence in caring for emergent patients [29]. The primary purpose of this project was to profile medical student reactions to their encounters with both high-fidelity and virtual reality simulation assessments using standard program evaluation surveys of our medical education program features. For this investigation, we involved all medical students from an entire medical school cohort, irrespective of their chosen specialty. A secondary purpose was to compare student competence in caring for seriously ill or injured patients (EPA-10) across both simulation platforms, using the entrustment decisions assigned by faculty observers.

## Methods

### Population of interest and sampling strategies

The population of interest was undergraduate medical students who were at the clinical clerkship level of medical school (typically third- and fourth-year students). Beginning in the academic year ending in 2022 we sampled the entire population of fourth-year students at our medical school during their required emergency medicine clinical clerkship. Students were only included in our study if they had consented to our using their evaluation and performance assessment data for research. Student consent is obtained from our college of medicine through written informed consent forms. This study was approved by The Ohio State University Human Subject’s Review Board on March 8, 2022 (Study #2022B0091).

### EPA-10 assessments

Our EPA-10 assessment program was initiated in 2016 and has been described elsewhere but is summarized here [13]. The EPA-10 assessment was designed as a capstone assessment of a fourth-year medical student’s competence in caring for an unstable patient with an acute emergent injury or pathology (EPA-10). These assessments were conducted at the conclusion of the student’s required emergency medicine clerkship using high-fidelity (HF) manikins in a simulated resuscitation bay. The assessment was summative in nature and contributed to the student’s clerkship grade. The instruments and cases previously developed for EPA-10 assessment using HF simulation have been demonstrated to have appropriate psychometric properties, such as content validity, internal consistency and inter-rater reliability [13, 14, 21, 27, 28].

For the original EPA-10 assessments, students were assigned to teams of three or four and provided two-hours of assessment time towards the end of their emergency medicine clerkship. To isolate each individual student’s performance while providing the realism of working in teams, each student was assigned as team leader for one case while the other students provided support. This way students see between three-four cases. Cases lasted about 30 min to run and debrief. Team leaders are the target of assessment and receive a score from a scoring rubric associated with the case, which includes a designation of entrusted if they successfully navigate all the critical actions in the simulated case without raising concerns from the instructor. Those not designated as entrusted are offered opportunities for remediation (see Supplementary Material 1). There are currently eight emergent patient (EPA-10) cases in our case bank that are randomly rotated over the course of an academic year.

During 2021–2022, we introduced a formative VR simulation of our EPA-10 assessments which we delivered one week in advance of the student’s summative HF assessment. Two of our cases (an ST-Segment Elevation Myocardial Infarction (STEMI) with ventricular fibrillation (Vfib) and a gastrointestinal (GI) Bleed) were converted to VR by SimX (SimX, Inc. San Francisco, CA.) for these assessments, which also used the Meta Quest 2 VR Headsets (Meta, Menlo Park, CA.). Prior to the VR encounter, students were instructed to watch an orientation video (https://www.youtube.com/watch?v=OCia8sHaxW4) that reviewed the basic mechanics and controls of the VR software. They also received a brief orientation during which they could explore and interact with the virtual environment before the scenario began. During the actual EPA-10 VR encounter, two students worked together in the same VR space and were observed and assessed by one faculty rater using a scoring rubric associated with the case (see Supplementary Material 1). Under both HF and VR conditions, the students received feedback on their performance from the faculty observer, however under the VR condition, feedback was formative. Students concerned about side effects from the VR, such as motion sickness, were able to opt out of the VR encounter and participate in an alternative experience which resembled an oral examination case. Only one student was unable to complete the VR encounter due to physical limitations. Over the study period, approximately eight different faculty facilitated the HF simulations and five of these same faculty facilitated the VR simulation sessions.

At the conclusion of the fourth-year EM Rotation students completed evaluations of the teaching faculty and of the rotation components, including the HF and VR encounters. The evaluations involved a single item on which students rated the activity as to how well it contributed to their medical education. The rating item used a five-point rating scale with 1 = poor, 2 = fair, 3 = satisfactory, 4 = very good and 5 = excellent. Students were also asked to provide responses to critical incident style questions such as “What part of the activity was done well and should be maintained in the future?” and “How can this activity be improved?”

### Data management and analysis

#### Quantitative analyses

We had 216 fourth year medical students participate in the emergency medicine clerkship during this study period. Of those 216 students, 184 (85.2%) consented for us to use their evaluation and assessment data from their EPA-10 assessments, and 160 (74.1%) returned at least one of the two evaluation surveys for the two assessment encounters, (Please note: students were eligible to complete an evaluation survey for HF, VR, or both encounters). We received 139 (64.4%) student evaluations for the VR encounters and 146 (67.6%) student evaluations for the HF encounters. We received both HF and VR surveys and were thus able to pair 125 (57.9%) of the evaluations for statistical and thematic analysis. We were able to pair 145 (67.1%) simulation outcomes (entrustment decisions). Seventeen students from this cohort were excluded from analysis because their VR simulations were canceled due to issues with the VR hardware.

Ratings of both HF and VR simulations were compiled and analyzed using SPSS (IBM Corp. Released 2021. IBM SPSS Statistics for Windows, Version 28.0. Armonk, NY: IBM Corp). To directly compare evaluations of students who completed both HF and VR simulation evaluations, we computed the difference in ratings by subtracting the HF rating from the VR rating (Difference = VR – HF) and analyzed these paired differences using the related-samples Wilcoxon Signed Rank Test. We compared platform ratings using Median Boxplots as a post hoc test to explain the Wilcoxon results and plotted difference scores over the year to see if ratings changed over time. Evaluation ratings were assumed to be ordinal level data, so we used these non-parametric statistics, Wilcoxon Signed Rank Test and Median Boxplots for this analysis. The categorical entrustment categories (Was the student designated as entrusted, yes or no?) were tallied for each assessment platform and compared using a McNemar Change Test for paired outcomes. (Vassar Stats, Richard Lowry, Vassarstats.net).

#### Summative content (thematic) analysis

We used summative content analysis to process the student comments into themes [32–34]. We assembled a preliminary coding table, comprised of common codes that are consistent with program evaluations then tested these codes by having two coders (MM and CS) code random samples of 25 comments from each of the HF and VR evaluations. The thematic coding table was subsequently modified to accommodate new codes added by the first two coders.

Using the revised coding table, the two coders (MM and CS) independently coded all comments from students who evaluated both the HF and VR platforms. A third coder (DW) resolved discrepancies between the first two, but also reviewed all codes for accuracy and checked to ensure that all codes were properly categorized as either strengths or weaknesses. The codes used for coding and minor modi­fications can be seen in Supplementary Material 2 (Old Codes). New code labels were applied so themes could be reported sequentially from most common to least common and as reported as strengths “S” or weaknesses (or needs improvement) “N” [35] (Supplementary Material 2).

## Results

### Quantitative results

Students performed significantly better in the HF than they did the VR simulation (*p*≤.001). Among the 145 students who did both simulations, significantly more students demonstrated competence or “entrustment” in their care of the critically ill patient through their performance in HF as compared to VR. All but a few students (91.7%) were rated as competent (attained entrustment) in HF, whereas only a little more than two-thirds (65.5%) of students demonstrated competence (attained entrustment) in VR (McNemar unsigned difference = 26.2, df = 1, *p*≤.001, Odds-Ratio = 5.75). The Cohen’s D effect size (es) of an odds-ratio this size is .96, which is considered a large effect [36].

**Figure 1.**
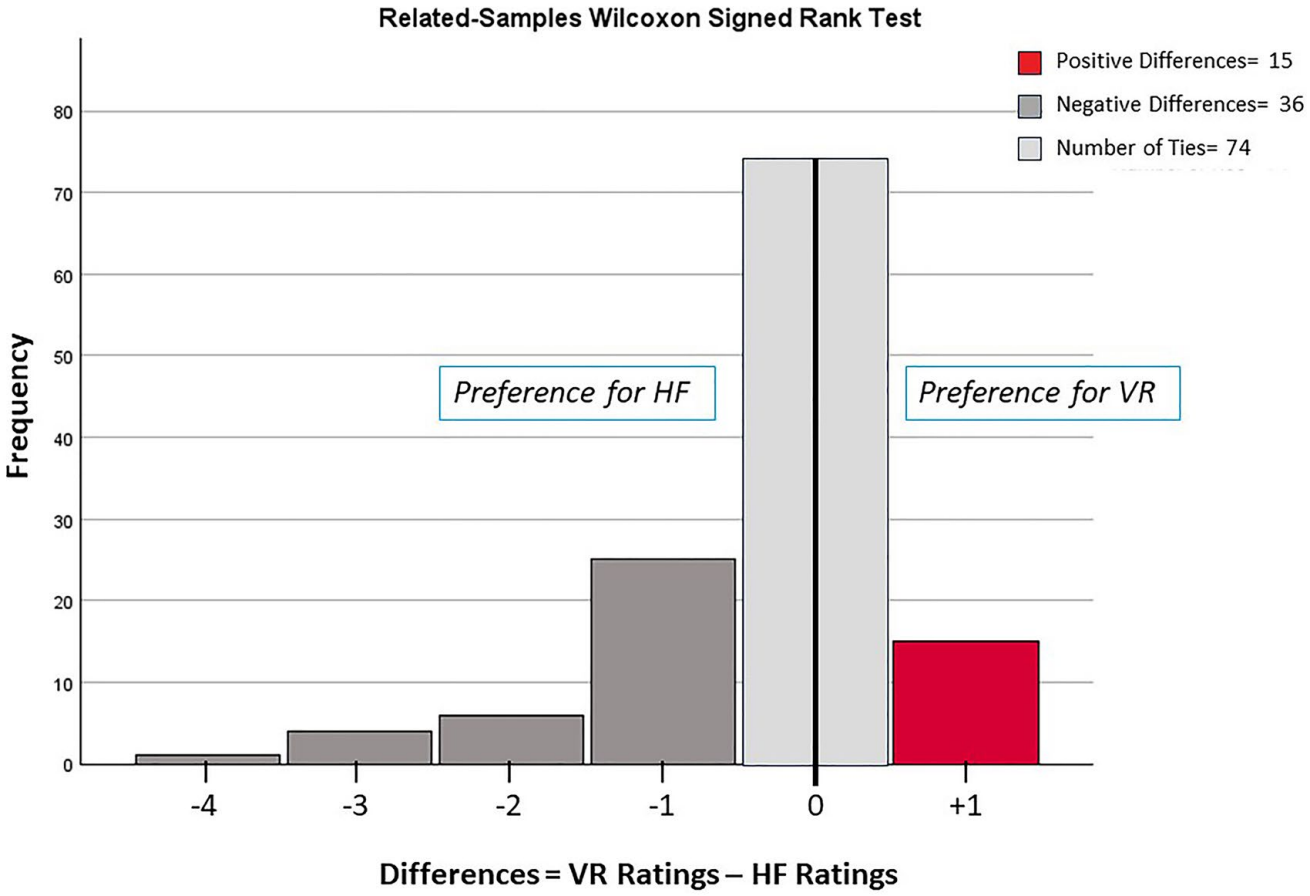
Frequency distribution of the differences in ratings generated by subtracting the rating of the high-fidelity simulation from the rating of the virtual reality simulation, a step in the Wilcoxon Signed Rank Test for related samples. Ratings were derived from the evaluation prompt: on a scale from 1–5, rate the quality of this session.

When comparing the ratings of the two simulation platforms, 28.8% (36 of 125) rated the high-fidelity experience higher, 12% (15 of 125) rated the virtual reality experience higher, and 59.2% (74 of 125) had no preference. The related samples Wilcoxon Signed Rank Test showed a significant difference between student preferences for an EPA-10 assessment platform, suggesting a slight preference for HF over VR platforms. (*T* = 337.5, *N* = 125, *p*=.001) (See Figure 1).

**Figure 2.**
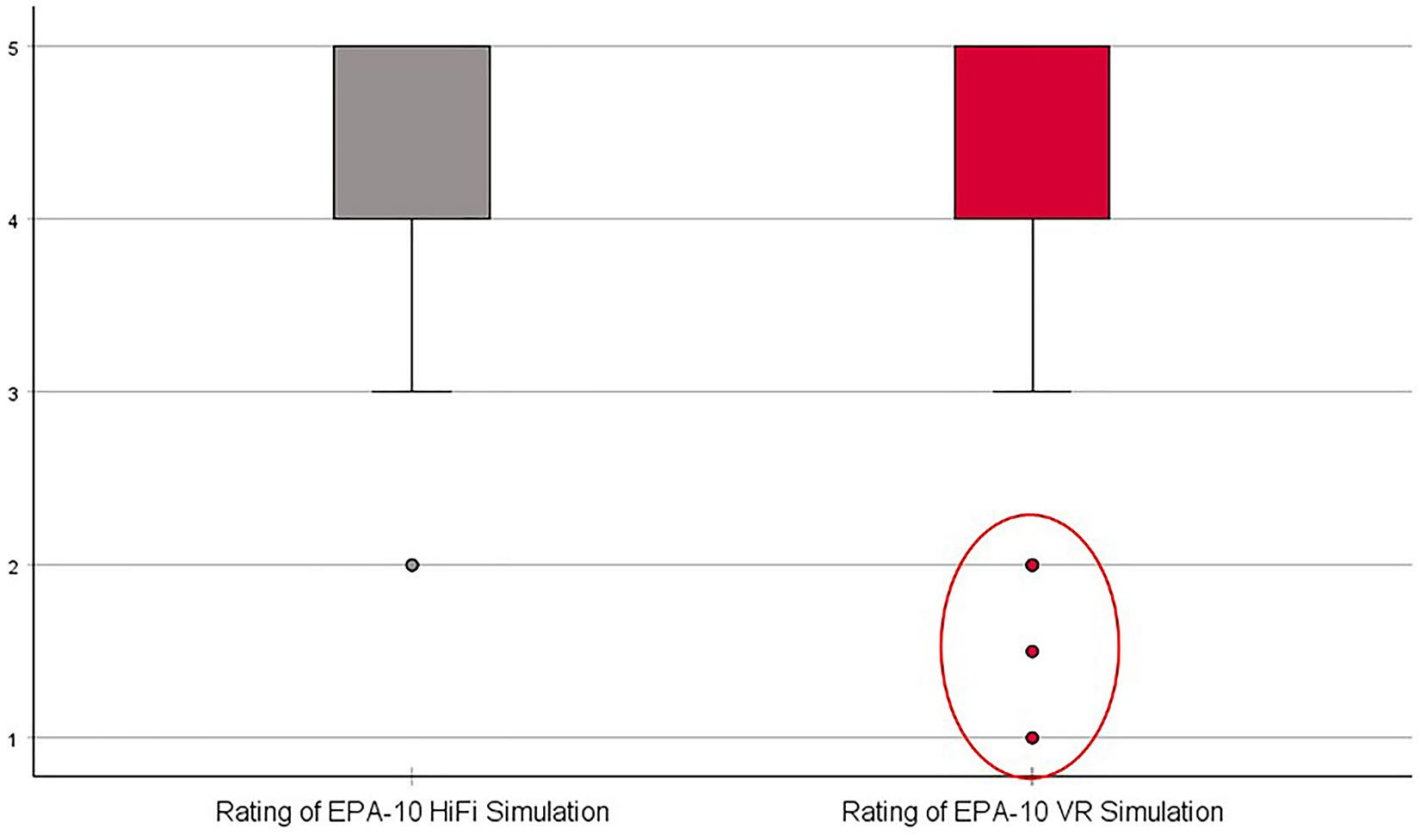
Boxplots comparing 128 fourth year medical student’s ratings of two simulation platforms, high fidelity manikin simulator and virtual reality simulator, for assessing EPA-10 skills: Managing the acute care patient in the emergency department [4].

The median boxplot analysis, which was used as a post-hoc test to explain the significant results of the Wilcoxon test, suggested that the distribution of ratings across both platforms were relatively comparable, except that the VR ratings distribution contained a cluster of low outlier ratings (Figure 2). These outliers explain the significant difference in ratings between the platforms observed in the Wilcoxon test results. The significant difference stems from the small number of students who were dissatisfied with their VR experience.

**Figure 3.**
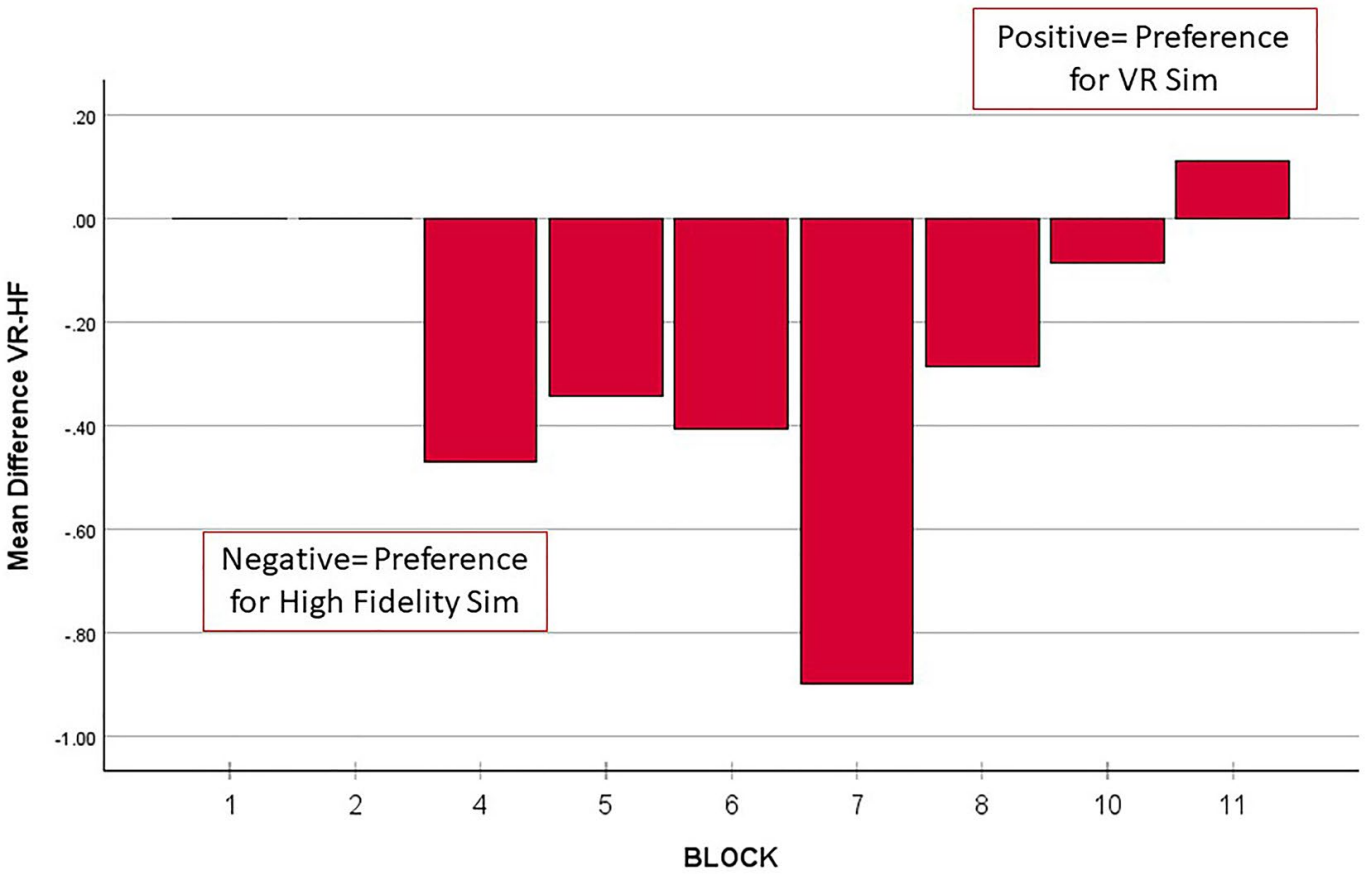
Bar graph of the mean differences in ratings of the two simulation platforms used for EPA-10 assessment. The difference in ratings is calculated by the formula: VR rating – HF rating, so that positive mean differences reflect a preference for VR and negative mean differences reflect a preference for HF.

Finally, we used the differences in ratings (Difference = VR – HF) to determine if student platform preferences changed throughout the year. We can then interpret positive values as a preference for VR and negative values as a preference for high-fidelity. A zero difference is interpreted as no preference. Figure 3 shows the mean difference between ratings (VR-HF) by rotation block over the course of the 2021–2022 school year.

Students from both Blocks 1 and 2 indicated no preference, as their ratings for both modalities were equivalent. These blocks were primarily comprised of students pursuing Emergency Medicine as a career. Between Blocks 4 and 10, however, students started to express a clear preference for the high-fidelity modality. As the academic year progressed, the students’ opinions began shifting towards a preference for VR (Block 11).

### Summative content (thematic) analysis results

Table 1 contains the frequencies of positive comment themes related to the strengths of the assessments by platform. Table 2 contains the frequencies of negative comment themes related to weaknesses or suggestions for improvement also presented by platform. Both tables provide the number of students making comments and the total number of comments made. Percentages in the tables are based upon the total numbers of comments made rather than number of students. We have also added Table 3 that contains illustrative comments that demonstrate examples of coding.

**Table 1.** Number and percentages of comments offered about strengths of the simulation platform: high fidelity or virtual reality.

		High-fidelity (HF)	Virtual reality (VR)	Total
Code-N	Definition	(*N* = 141)	(*N* = 160)	(*N* = 301)
S1	Debriefing, feedback, or teaching from instructor during simulation was good; contributed to my learning	110 (42.0)	76 (25.4)	186 (33.2)
S2	Generally positive comments or expressed enthusiasm for experience	26 (9.9)	39 (13.0)	65 (11.6)
S3	Patient cases were good, appropriate, helpful, or related to content for which we prepared	24 (9.2)	18 (6.0)	42 (7.5)
S4	Session was well conducted or facilitated	20 (7.6)	20 (6.7)	40 (7.1)
S5	Fun, cool, interesting, unique way of learning and practicing	5 (1.9)	34 (11.4)	39 (7.0)
S6	We were well prepared for encounter with instructions, pre-briefing orientation and instructional videos	5 (1.9)	28 (9.4)	33 (5.9)
S7	Simulation was hands-on, immersive, realistic setting, contributed to effective learning of concepts	16 (6.2)	16 (5.5)	32 (5.8)
S9	Request for more opportunities to do additional sessions/cases/experiences (because the experience is valuable)	14 (5.3)	15 (5.0)	29 (5.2)
S10	Simulation was good preparation for clinical practice	10 (3.8)	15 (5.0)	25 (4.5)
S11	The VR experience was a good preparation for HF session		21 (7.0)	21 (3.7)
S12	Simulation environment was a positive learning environment, (Students felt some stress, but also safe)	12 (4.6)	6 (2.0)	18 (3.2)
S13	Appreciate opportunity to lead, experience teamwork	11 (4.2)	3 (1.0)	14 (2.5)
S14	Prefer HF practice to VR	3 (1.1)	8 (2.7)	11 (2.0)
S15	Repetition in HF (with four cases) helped provide the practice needed to improve	3 (1.1)		3 (0.5)
S16	Nurse confederate facilitated communication	2 (0.8)		2 (0.4)
S17	Helped with consultant communication	1 (0.4)		1 (0.2)
Total comments		**262**	**299**	**561**

N = number of students who offered comments. Please note: because students could offer more than one comment, percentages are calculated using the total number of comments as the denominator rather than total number of students.

**Table 2.** Number and percentage of comments offered about the weaknesses of the simulation platform: high fidelity or virtual reality.

		High fidelity (HF)	Virtual reality (VR)	Total
Code	Definition	(*N* = 141)	(*N* = 160)	(*N* = 301)
N1	More practice with simulators or simulation environment before the simulation encounter	14 (18.2)	37 (31.6)	51 (26.2)
N2	More guidance on roles and expectations related to the simulation encounter (better explanation of what to expect and how to prepare)	19 (24.7)	15 (12.8)	34 (17.6)
N3	Technical problems with the simulators prevented positive experience	3 (3.9)	22 (18.8)	25 (12.8)
N4	Ineffective or insufficient debriefing session or feedback after simulation encounter	15 (19.5)	9 (7.7)	24 (12.4)
N5	Time management: poor timing of the cases, rushed session, session ran over	6 (7.8)	7 (6.0)	13 (6.7)
N6	Simulation provoked motion sickness, disorientation, or anxiety	1 (1.3)	11 (9.4)	12 (6.2)
N7	Poor preparation for the cases covered in the simulations (medical content, procedures, were unfamiliar)	6 (7.8)	3 (2.6)	9 (4.7)
N8	Fidelity of the simulator was too low; simulation was not realistic	5 (6.5)	2 (1.7)	7 (3.6)
N9	Expectations were unrealistic	3 (3.9)	1 (0.9)	4 (2.1)
N10	Grading of encounter was unfair: cases were of unequal difficulty. Order gave advantage to those who went last. No credit for team members who were not leads.	3 (3.9)	1 (0.9)	4 (2.1)
N11	Poor timing during the clerkship	2 (2.6)	1 (0.9)	3 (1.6)
N12	Need a nurse confederate in VR to assist with case		2 (1.7)	2 (1.0)
N13	VR was NOT good preparation for the high-fidelity simulation		2 (1.7)	2 (1.0)
N14	Session was poorly organized, communicated (or run)		1 (0.9)	1 (0.5)
N15	Desire for more case diversity or for specific types of cases (one of the scenarios should have been a code)		1 (0.9)	1 (.05)
N16	Environmental challenges (the room was hot)		1 (0.9)	1 (0.5)
N17	Concern with equipment hygiene (cleanliness of equipment)		1 (0.9)	1 (0.5)
Total comments		**77**	**117**	**194**

N = number of students who offered comments. Please note: because students could offer more than one comment, percentages are calculated using the total number of comments as the denominator rather than total number of students.

**Table 3.** Illustrative comments related to the strengths and weaknesses of simulation for learning and assessing EPA-10.

Strength code	Weakness code	Illustrative comment
S2, S11		I thought it was a really interesting experience to use the virtual reality simulations at least once before graduation. I think education will continue to evolve and use more of these systems so I appreciate having some exposure. (S2) It was also helpful for preparing for our graded simulations the next week. (S11)
S1, S2, S9, S12		[The facilitator] provided very helpful, constructive feedback and clinical insight during the debrief session (S1). This was a very helpful session. (S2) It provided learners practice engaging in an emergency situation with colleagues (S9) while obviously having it be very low stakes, and without it jeopardizing patient safety or being overly traumatizing for the learner (S12).
S11, S7, S13, S6, S1, S2		I really appreciated this practice run before the actual code assessment next week (S11). The simulation was very realistic (S7) and I appreciated the collaborative approach (S13). I appreciated the tutorial video before the session (S6) and [the facilitators] appropriate feedback afterwards during the debrief (S1). This has been developed very well and is an asset to our rotation. Thank you for organizing this session. It was very helpful for my learning and growth in assessing emergent situations. (S2)
S9, S1		This session was good practice for leading codes/emergency room evaluations since we don’t get much direct experience with higher acuity things in the ER as med students (S9). [The facilitators] feedback afterward was really helpful! (S1).
S5, S2, S8, S1		Wow! great fun! (S5) and great educational value. (S2) Wish this was integrated more extensively throughout the curriculum (VR set for every student?? if only) (S8). Great debrief after the sim (S1).
S2, S9, S8		This was one the of the best educational experiences I have had as a fourth-year medical student (S2). In no other setting can you apply and practice your medical knowledge and skills in a system that allows for learning critical decision-making skills (S9). This should be standard training for all fourth-year medical students as preparation for intern year (S8).
S2, S7, S4, S1	N3	Despite some technical difficulties (N3), I thought this session was great! (S2) I appreciated that the simulation was able to give real feedback on what was going on, and allowed the student to take a more active role in caring for the patient compared to other virtual scenarios I’ve completed in medical school (S7). [The facilitator] was accommodating to students. (S4) He answered all questions clearly, provided advice for exam preparation, and provided valuable feedback on the case (S1).
	N2, N7, N6, S14	Session could have had a better explanation or instructions for preparation (N2). Was unsure how to prep for the actual knowledge we would be assessed on due to a lack of information on what the session would contain (N7). Additionally, the VR made me as well as many other students extremely nauseous, dizzy, and lightheaded (N6). Would have much preferred to just do this session in person (S14).
S5, S9	N6	I personally have never experienced a VR system previously but it was an interesting experience (S5). I also have never had a panic attack but I imagine this is how they feel. I believe with the weight of the headset and the mask, it felt like I had a bag over my head and I felt (very) uncomfortably restricted not knowing my surroundings (N6). I guess this is more of a personal issue than one regarding the session. I appreciate that we had this experience prior to our actual tested sim (S9).
	N1, N3, N6	I think the VR would be more helpful with multiple sessions (N1). Also my VR headset had the floor flashing with alternating stripes of wood planks and white for the whole duration of the simulation, which was pretty distracting and made me feel a little dizzy (N3, N6). It was also hard to read some of the fine details in the VR headset because it would be blurry even with adjusting the angle of the headset, like the EKG or lab values (we couldn’t tell if something was a comma or a period and that difference affected whether it was a normal/abnormal lab result) (N3).

#### Strengths

Interestingly, students offered nearly three times the number of comments regarding strengths of EPA-10 simulations as they did weaknesses. The themes of positive comments were consistent across platforms. Comments regarding the one-on-one teaching that occurred during simulation encounters were the most frequently offered across both platforms. While this represents 33.2% of all comments made (186 of 561), affirmation of the teaching that takes place during simulation sessions through debriefing, feedback or coaching was made by 62% (186 of 301) of all students. Another unsolicited comment that was frequently made had to do with general praise for the simulation experiences. Nearly 22% (65 of 301) students expressed gratitude or appreciation for the opportunity afforded them to experience these simulations of EPA-10 cases.

We noted three major differences between the number of comments offered across platforms. The first involved student’s enthusiasm for the VR platform, through generally positive comments (13% or 39 of 299) or through the use of enthusiastic terms such as “fun, cool, or interesting” to describe their VR experience (11% or 34 of 299). HF also received generally positive comments (10% or 26 of 262), and enthusiastic ones (2% 5 of 262), but not as many as VR. A second difference involved the number of comments received about how well-prepared students were for the VR encounter. A little more than 9% (28 of 299) of the comments fell into this category, while only 1.9% (5 of 262) of these comments were made about preparation for HF. Finally, over 7% (21 of 299) of the comments were directed at how well the VR experience prepared students for the HF simulation that occurred a week later.

#### Weaknesses

Except for the fact that a number of students wanted better explanations of what to expect and how to prepare for the simulations, the weakness comments were not generally consistent across cohorts. Nearly a third (31.6%, 37 of 117) of the comments made about VR were directed towards a request for more practice with the simulator before the encounter. For HF, only 14 of 77 (18.2%) of comments were made relating to more practice. The VR platform received substantially more complaints about technical issues (18.8%, 22 of 117) than did HF (3.9%, 3 of 77). Table 3 shows some of the common comments received by students who had complaints about technical issues. A higher percentage of the comments received about the HF encounter as compared to the VR encounter had to do with students needing more guidance on roles and expectations. The same was true regarding complaints about ineffective debriefing.

## Discussion

We set out to accomplish two objectives with this investigation. First, we hoped to learn how students perceived our simulation programs for assessing EPA-10 skills, both the HF and VR platforms. Second, we hoped to compare student performance across the two platforms through faculty observers’ entrustment ratings. The most important finding of this study was that significantly and substantially fewer students demonstrated “entrustment” in their care of the critically ill patient through their performance in VR as compared to HF. Only a little less than two-thirds (65.5%) of students demonstrated competence (attained entrustment) in VR, whereas nearly all students (91.7%) were rated as competent (attained entrustment) in HF. While we recognize that numerous differences between the two simulation platforms exist, this finding suggests that the use of the VR platform for summative assessment is currently limited.

As anticipated by our summative content analysis (thematic) coding table (Supplementary Material 2), students either offered praise or raised concerns about simulation platforms based on the foundational elements of Brain-based Learning (BBL) Theory [15–16]. Students commonly commented on whether the simulation was immersive and realistic and whether they were sufficiently acclimated to the simulation environment. These comments reflect student concerns about the BBL foundational elements of relaxed alertness, or whether the setting was immersive and realistic, but free of stress [15]. Students’ enthusiasm for these simulation platforms might also be explained by the BBL element orchestrated immersion, which suggested that they were fully engaged in the simulation exercise [15–16]. A few students expressed dismay with their VR experience due to feelings associated with motion sickness, however all were able to complete the encounter. Recent improvements to the visual fidelity of head mounted displays may reduce the number of these types of complaints about motion sickness in VR. (Meta Quest 3 VR Headsets (Meta, Menlo Park, CA.)).

Similar evaluation research by McNamara, et al. resulted in similar findings, that student experiences with VR were rated similarly to their experience with HF [29]. Students in both studies found VR to be an enjoyable and immersive experience and believed that VR was a sufficiently realistic environment for learning. Students in both studies also expressed a slight preference for HF as a learning experience and as a platform for learning to work in teams. Additionally, both studies suggested that VR platforms are more challenging and less intuitive to use than HF platforms.

Because our study involved six times more students preparing for residency in more than one specialty, we also observed some key differences to the McNamara study [29]. Comparatively, our students commented more frequently on the value of the VR encounter and how it contributed to their learning, either by preparing them for their anticipated HF performance assessment or for clinical practice in the future. Additionally, our students commented far more frequently on the value of feedback and teaching received during both types of simulation encounters. Finally, McNamara and colleagues did not study or discuss performance outcomes across the two simulation platforms, so we are unable to conclude whether our observed differences are the result of platform, or the result of the rules of engagement: formative vs. summative; 2-person vs. 4-person teams; or nurse confederate help vs. no help; and finally whether the repetition effect of participating in three-four HF encounters vs. only one VR encounter [29].

We speculate that fewer students were rated as competent (met entrustment) in VR than in HF for a variety of reasons. The primary reason involved the nature of the assessment; the VR assessment was intended to be formative, so that the student could get a feel for running an EPA-10 case and get feedback on their performance. The HF encounter was intended to be a summative assessment that contributed to their overall grade for the clerkship. By their very nature, the formative assessment is low-stakes and offers a more relaxed environment than a high-stakes summative assessment. However, other differences between the two platforms also likely contributed to the difference in performance. First, students seemed to need more practice or experience in acclimating to the VR simulation environment as compared to the HF environment, which more closely resembled a real trauma bay. Second, in VR, we required the students to perform all the tasks related to caring for the seriously ill patient, whereas in HF, the students were able to request help from a nurse confederate. The increased cognitive load required to perform the additional tasks likely contributed to lower performance. Third, the HF simulation was a much longer experience since students completed 3–4 cases in it, as opposed to only a single case in VR. This may have contributed to students having more comfort in HF simply because their engagement with it was prolonged. Finally, student teams in VR involved only two students working together, while in HF the teams were comprised of either three or four students.

VR simulation can be offered more often and at a much lower cost than a comparable HF simulation, increasing its potential for use as a training platform in addition to its use for assessment. Furthermore, the VR headsets and the gaming computer used to run them can be used for other types of simulations. Currently, due to patient safety concerns, medical students are not afforded the opportunities to participate in the diagnosis and management of high acuity patients. Since the VR simulations can be offered more often and at much lower cost than comparable HF simulations, they are well suited for use as a training platform for EPA-10 skills, particularly as a practical environment for the deliberate practice needed by learners to master a skill [37].

### Limitations

We confronted numerous technical and logistical challenges related to offering EPA-10 assessments to our students. Besides the logistical challenges of staffing these assessments with qualified faculty instructors, which were a challenge for both assessment platforms, our simulations were also interrupted by the pandemic and inclement weather. Consequently, a substantial number of our students were unable to participate in either the HF or VR simulations or both. We also confronted more technical challenges with our VR assessments, causing inconsistent experiences for students over this first year of implementation.

The two platforms were intended to use the same cases, consist of environments that appeared to be similar, and be constructed to require the same performance tasks. Unfortunately, however, there were differences between platforms that limit their comparability. One limitation involves placing two students in the VR who must share responsibility for patient care. This makes assessment more challenging. Another involves our ability to provide a nurse confederate in VR, requiring the students to perform their own nursing tasks. These limitations related to the VR platform suggest that as currently configured, their use for summative assessment of EPA-10 skills is limited.

### Future research efforts

We believe that consistent with BBL Theory [16], both HF and VR simulation platforms serve to provide a constructive learning and assessment environment for EPA-10 skills. Future studies that would contribute to updating the literature should most certainly involve controlled experiments in order to inform medical educators more about the importance of various simulation platform features: such as the ability to work in teams, the need for a nurse to perform nursing tasks, and the impact of assessment decisions on performance (whether summative or formative). Another potential line of research involves cost comparisons and cost benefit analysis to better inform medical educators on how to offer simulations that they can afford to their medical students.

## Conclusion

Once our VR simulation platform was functioning without technical problems, it served as an acceptable simulation platform for delivering formative assessments of EPA-10 skills to our medical students. More rigorous studies of VR will be needed before it can be justified as a simulation platform for summative assessment. The strength of VR is its potential for delivering deliberate practice and formative feedback so that learners are able to master clinical skills, at a relatively low cost, giving medical educators more tools to deliver instruction on content that is challenging to deliver in the clinical environment with live patients. Using VR simulations, medical learners can obtain experience in confronting cases that portray critically ill or injured patients without jeopardizing patient safety. VR educational activities should be designed to capitalize on intrinsic motivation, mastery goal orientation, and achievement emotions to improve learning [24].

When introducing VR as an assessment platform, medical educators must also be aware that learners require opportunities to acclimate to the VR environment. Prior to any form of assessment, they should have the opportunity to practice using the controllers to navigate the environment and manipulate tools in the virtual space. Until the technology matures, VR instructors should be mindful of potential technical challenges. Furthermore, instructors ought to screen for susceptibility to motion sickness in advance of student participation in the VR environment. Finally, instructors should treat simulation platforms just as they do bedside teaching, as an opportunity to deliver high-quality feedback on the learner’s performance, as students identify this aspect as the most important component of the experience.

## Supplementary Material

Supplemental Material

## Data Availability

The data that support the findings of this study are available upon request from the corresponding author, David Way, MEd. The data are not otherwise publicly available due to containing information that could compromise the privacy (FERPA) of research participants.

## Draft manuscript preparation

MM, DW, CSM. All authors reviewed the results and approved the final version of the manuscript.
