# Supplementary material for: Evaluation of high-fidelity and virtual reality simulation platforms for assessing fourth-year medical students’ encounters with patients in need of urgent or emergent care
Source: Ann Med. 2024 Jul 30;56(1):2382947. doi: 10.1080/07853890.2024.2382947 (PMC11290289; doi:10.1080/07853890.2024.2382947)
Supplement: Supplemental Material [file IANN_A_2382947_SM6664.zip › Supp_Data/Appendix 1 EPA10 Score Rubric Revised Clean.docx]

Appendix 1. Scoring Rubric for EPA-10 Patient Encounter involving an ST-elevation myocardial infarction (STEMI).

EPA-10 Assessment – STEMI Case

1. Performs a focused history and physical exam (including obtaining vital signs)

- No
- Incorrectly or incompletely
- Yes, with prompting
- Yes

2. Obtains an EKG

- No
- Incorrectly or incompletely
- Yes, with prompting
- Yes

3. Activates Cath Lab (Ok if STAT Cards consult if they explicitly state concerned for STEMI)

- No
- Incorrectly or incompletely
- Yes, with prompting
- Yes

4. Gives 324 mg oral ASA

- No
- Incorrectly or incompletely
- Yes, with prompting
- Yes

5. Initiates and continues chest compressions without significant pauses

- No
- Incorrectly or incompletely
- Yes, with prompting
- Yes

6. Promptly performs rhythm check and recognizes rhythm as vfib

- No
- Incorrectly or incompletely
- Yes, with prompting
- Yes

7. Delivers defibrillation of 200 J or more

- No
- Incorrectly or incompletely
- Yes, with prompting
- Yes

8. Did this student perform any dangerous actions?

- No
- Yes

**9. Entrustment Statement: Please rate your entrustment of this student according to these instructions. The student must have a “Yes” response *“without prompting”* for all critical actions. (Items ).**

Would you feel confident in this student’s ability to manage an acutely decompensating/acutely ill patient with a life-threatening illness?

- No
- Yes

10. Additional Comments Regarding EPA 10
