# Supplementary material for: Evaluation of high-fidelity and virtual reality simulation platforms for assessing fourth-year medical students’ encounters with patients in need of urgent or emergent care
Source: Ann Med. 2024 Jul 30;56(1):2382947. doi: 10.1080/07853890.2024.2382947 (PMC11290289; doi:10.1080/07853890.2024.2382947)
Supplement: Supplemental Material [file IANN_A_2382947_SM6664.zip › Supp_Data/Appendix 2 Revised Clean.docx]

**Appendix 2. Coding Table for Thematic Coding**

| **OLD CODE** | **NEW CODE** | **THEME** |
| --- | --- | --- |
| 1 | S4 | Session done well/facilitated well |
| 2 | S1 | Good Discussion/Debrief/Feedback/Teaching |
| 3 | S12 | Simulation environment was a positive learning environment, (Students felt some stress, but also safe) |
| 4 | S3 | Good Case/helpful cases/appropriate content/prepared for content |
| 5 | S16 | Positive Nurse confederate to aid communication |
| 6 | S7 | Concepts into practice/hands on/immersive/realistic |
| 7 | S5 | Fun, cool, interesting, modality unique |
| 8 | S17 | Consultant Communication |
| 9 | S6 | We were well prepared for encounter with instructions, pre-briefing orientation and instructional videos. |
| 10 | See N1 | Additional High-Fidelity practice (to help prepare for sim) USE 13 INSTEAD |
| 11 | N11 | Poor timing during the clerkship |
| 12 | S8 | Request for more opportunities to do additional sessions/cases/experiences (because the experience is valuable) |
| 13 | N1 | More practice with simulators or simulation environment before the simulation encounter |
| 14 | N2 | Unclear role/expectations, more explanation of session |
| 15 | N9 | Unrealistic expectations |
| 16 | N4 | Bad debrief/bad feedback/specific improvements for debrief |
| 17 | N8 | Fidelity of the simulator was too low; Simulation was not realistic |
| 18 | N3 | Technical Challenges |
| 19 | N7 | Poor Preparation (medical knowledge, content, unable to study) |
| 20 | S11 | The VR experience was a good preparation for high fidelity session |
| 21 | S9 | Simulation was good preparation for clinical practice |
| 22 | S13 | Appreciate opportunity to lead, experience teamwork |
| 23 | S14 | Prefer HF practice to VR |
| 24 | N10 | Unfair to go first (USE 13 if learning issue or 27 if related to grading) |
| 25 | N5 | Poor timing of the cases, rushed |
| 26 | N6 | Motion sickness/disorienting/anxiety provoking |
| 27 | N10 | Grading issues: Some Cases harder than others/unfair grading for single case, Unfair to go first (From Code 27), Grading should be limited to team leader (From Code #32), Should not be graded, rather it should just be (formative) learning experience (From Code #24) |
| 28 | N12 | Request for nurse confederate in VR |
| 29 | N16 | Environmental Challenges (the room was hot) |
| 30 | N13 | VR not good preparation for the high-fidelity sim |
| 31 | N17 | Cleanliness concern |
| 32 | N10 | Grading shouldn't be limited to the team leader, should just be a learning experience |
| 33 | N15 | Specific content recommendations eg. I wish one of the scenarios would have been a code |
| 34 | S2 | Generally positive (nothing specific) |
| 35 | N15 | Direct comparison between HF and VR |
| 36 | N14 | Session was poorly organized |
| 37 | S15 | Repetition in the HF (with 4 cases) helped provide practice needed to improve. |
